# Knowledge and attitudes toward over-the-counter medications among pharmacy students: insights from a cross-sectional study in Taif University, Saudi Arabia

**DOI:** 10.3389/fmed.2024.1435707

**Published:** 2024-11-12

**Authors:** Mohammed S. Alharthi, Hassan Almalki, Faisal Alsubaie, Fawaz Alotaibe, Abdullah Abuasiah, Faisal Basha, Mohammed M. Aldurdunji, Nasser M. Alorfi

**Affiliations:** ^1^Department of Clinical Pharmacy, College of Pharmacy, Taif University, Taif, Saudi Arabia; ^2^Department of Pharmaceutical Practices, College of Pharmacy, Umm Al-Qura University, Makkah, Saudi Arabia; ^3^Department of Pharmacology and Toxicology, College of Pharmacy, Umm Al-Qura University, Makkah, Saudi Arabia

**Keywords:** over-the-counter medications, self-medication, pharmacy education, health knowledge, students’ attitudes, and knowledge

## Abstract

**Introduction:**

Over-the-counter (OTC) medications are those obtained without a medical prescription from a healthcare professional. With the increasing availability of information from various sources, including social media, pharmacy students may be exposed to unreliable or inaccurate data. Incorrect medication use is particularly concerning due to its potential risk of causing adverse health effects.” Hence, this study aims to determine students’ knowledge and attitudes at Taif University’s pharmacy college.

**Methods:**

This research utilized a cross-sectional online questionnaire-based study, employing data from a sample of 450 pharmacy students from Taif University in Saudi Arabia. Descriptive analysis included descriptive and differential analysis. The data were analyzed using statistical package for social sciences (SPSS) Version 27.

**Results:**

The majority of participants, 297 (88.2%), were aware that inappropriate use of over-the-counter medications might have negative implications. A total of 233 participants (51.8%) reported having previously used an OTC medication. Also, 293 (65.1%), were aware that using OTC medications beyond their expiration date was harmful. A total of 280 participants (62.2%) had a high knowledge of OTC medication, whereas 170 respondents (37.8%) had a low level of knowledge. A significant correlation was found between age, year of study, and the use of OTC medication *p*-values <0.05 (0.016*, 0.003*, and 0.001* respectively) and understanding of OTC medication. Gender had no significant influence on knowledge of OTC (*p* > 0.05).

**Conclusion:**

The study found positive attitudes toward OTC medications. Due to increased pharmaceutical exposure and self-medication, upper-year students and OTC course graduates comprehend OTC medications better. The examination found safety protocol violations in expiration dates, prescription label interpretation, and storage. Therefore, the study provides useful information for future attempts. Also, this study may contribute to the literature and guide future research to fill knowledge gaps.

## Introduction

Over-the-counter (OTC) medications are medications that can be obtained from pharmacies or internet websites without the need for a prescription from a health care professional (1). OTC medications are necessary for treating and/or preventing minor medical ailments such as muscle discomfort, headaches, allergies, and heartburn (2). Noncompliance with prescription medications, behavior of medication sharing, and the phenomenon of self-medication (acquiring medications without communicating with healthcare experts) are all important aspects of medication consumer behavior (3). The use of OTC medications for self-medication is a global health care occurrence driven by social and economic factors and is acknowledged as a common practice by the world health organization (WHO) worldwide than prescription-only medications (4–7). It demonstrates the importance for individuals to take ownership of their well-being and make their own decisions regarding which medication to take. OTC medications are a readily available type of self-care that allows a patient with symptoms to self-diagnose and treat promptly (8). Recent studies reported that there is a misguided belief that OTC medications are generally safe. The researchers in those studies revealed that OTC medications should be used with caution, adhering to manufacturer’s instructions to prevent the risk of serious side effects. Some studies found that controlling OTC medications is challenging for most countries (9–11). On a global scale, the misuse of OTC medications has increased and has been identified as a public health concern (11). Improper use of OTC medications refers to their use without a valid medical indication (12).

Furthermore, there have been recent reports that various analgesics and OTC medications have been abused by university students (10, 13). In such cases, self-medication may result in potentially inappropriate practice and health problems. Insufficient knowledge can lead to the administration of incorrect medications or dosages (14). Several researchers have reported that university students lack knowledge about OTC medications and have a poor attitude toward their proper use (15–17). Ultimately, this may lead to health issues, including adverse drug events, drug interactions and overdose (15–17). Among the OTC medications, Paracetamol is the most frequently administered analgesic by medical students and one of the most commonly self-prescribed medications (18). One study, which included 1,596 students (51.9% of whom were university students), was undertaken in Saudi Arabia to investigate the use of OTC medications among students during exams (19). According to the study, 80% of students used nonsteroidal anti-inflammatory drugs (NSAIDs) to manage pain, and female students were more likely to take OTC medications. Moreover, the National Hospital Morbidity Database (NHMD) in Australia reported that young adults are at high risk of paracetamol-related harm (20), with a total of 4,577 toxic cases reported involving paracetamol in 2017 and an overall increase in hepatic toxicity incidents between 2007 and 2017. To date, no study has investigated the knowledge and attitude of pharmacy college students regarding OTC medications at Taif University in Saudi Arabia, so this study was undertaken to investigate this cohort of students. Although OTC medications are widely utilized, the adequacy of OTC-related education in pharmacy programs across Saudi Arabia remains insufficiently explored. Empirical evidence indicates that gaps in knowledge and inappropriate attitudes toward OTC use can contribute to unsafe practices, including improper self-medication and medication misuse, especially among young adult populations. Given that pharmacy students represent future frontline healthcare professionals, their proficiency in OTC medication management is critical for ensuring patient safety and promoting evidence-based practice. Thus, addressing these educational gaps is essential to strengthen the curriculum and equip students with the necessary competencies to guide patients in the safe and effective use of OTC medications. In Saudi Arabia, pharmacy students undergo structured practical training, including mandatory internships in community and hospital settings. This supervised ‘Experiential Year’ enhances clinical skills, patient counseling, and familiarity with OTC medications. These internships bridge theoretical knowledge with practice, providing hands-on experience before graduation (21). The Saudi Commission for Health Specialties (SCFHS) outlines specific training requirements to ensure pharmacy graduates are well-prepared for professional roles which may influence their knowledge and attitudes toward OTC medications. Pharmacy education in Saudi Arabia begins with basic sciences in the first 2 years, followed by clinical practice courses in the third and fourth years, and intensive clinical rotations in the final years (22). The study’s findings could inform the development of targeted student training and continuing education initiatives for pharmacy professionals. This study aims to measure the knowledge and attitude of pharmacy college students at Taif University toward OTC medications.

## Materials and methods

### Study design

The present study employed a cross-sectional design involving pharmacy students from Taif University, conducted between September 2023 and April 2024. The inclusion criteria involved male and female students with internet access who could participate in this study, including students from year one to year six. The exclusion criteria were students outside the College of Pharmacy at Taif University and students who could not access the internet.

### Sampling and sample size

The study adopted a snowballing sampling technique. Snowball sampling was used to reach a diverse group of participants when direct access to the entire student population was challenging. To determine sample size, the authors used the Cochrane sample size approach, which is *n* = *Z*^2^(1 − p)/d^2^. Where n is the sample size, *Z* is the critical statistic for a 95% confidence interval, which is the anticipated proportion, which was set at 50%, and d is the margin of error, set at 0.05. The minimum acceptable sample size was 384, but the researchers adopted the sample size of 450 respondents to avoid non-response risk. The inclusion of students from diverse academic years and backgrounds captures a comprehensive view of the target population. To address potential sample heterogeneity, key variables such as academic year and gender were carefully considered during data interpretation. This approach strengthens the validity of the findings by ensuring that subgroup variations are appropriately acknowledged.

### Data collection and piloting

The questionnaire was designed to align with the study’s objectives. The questionnaire was developed following previous literature (1–4) and validated by a group of the academic staff of the College of Pharmacy; however, the questionnaire had been adjusted according to the study’s aim and objectives with the expert adviser and questionnaire piloting to obtain a particular context. The questionnaire addressed three main areas: demographic data, knowledge, and attitudes of students concerning OTC medications. For piloting, the questionnaire was sent to 10 students, and the ultimate goal of this pilot is to verify the questionnaire’s feasibility, understandability, and readability to ensure the questionnaire’s effectiveness. It was distributed online to the students through their mobile group texting software (WhatsApp® and Telegram®).

### Data analysis

After the data collection period, data was entered into Excel® spreadsheets. The data was coded and analyzed using the Statistical Package for the Social Sciences (SPSS) software version 27.0. Descriptive analysis included calculating frequencies with percentages, and means were used. Correlation analysis was conducted to identify any significant correlations between variables. A *p*-value of less than 0.05 was deemed statistically significant.

### Ethical considerations

Ethical approval was obtained from Taif University’s Committee for this study, application number 45–107. Among the key ethical considerations were confidentiality and informed consent. Before agreeing to participate, the participants were fully informed about the study’s purpose, procedures, risks, and benefits. Participants should provide voluntary and informed consent without coercion. Additionally, the researchers safeguarded participants’ privacy by protecting their identity and sensitive information.

## Results

Table 1 shows the summary of the demographic information. A total of 450 participants completed the online questionnaire. A substantial proportion, 170 (37.8%) of participants, were aged between 18 and 20, with nearly half (52.2%) being females. Regarding the year of study, students were distributed almost more or less the same across the 6 years of study, with a majority, 93 (20.7%), being in the fourth year of study.

**Table 1:** Demographic information of the participants (*N* = 450).

Demographic information	Category	Number and percentage *n* (%)
Age (years)	18–20	170 (37.8%)
	21–22	153 (34.0%)
	23–25	119 (26.4%)
	Over 25	8 (1.8%)
Gender	Male	215 (47.8%)
	Female	235 (52.2%)
Year of study	1st Year	91 (20.2%)
	2nd Year	67 (14.9%)
	3rd Year	48 (10.7%)
	4th Year	93 (20.7%)
	5th Year	70 (15.6%)
	6th Year	81 (18.0%)

### Participants’ knowledge about over-the-counter (OTC) medication

The vast majority, <80% of the participants, provided the correct definition of OTC medication and were aware that OTC medication could cause side effects when used improperly. While around 70% of the participants were aware that it is not necessary to use OTC medications that are prescribed by the physician. Furthermore, around 50% of the participants indicated that they had taken a course specifically about OTC medication. The majority, 293 (65.1%), knew that using OTC medication past its expiry date was unsafe and harmful if misused 413 (91.8%) and that they could not refer them to friends or family based on their personal use 232 (51.6%). A significant majority, 344 (76.4%), were aware that OTC medications are commonly used to treat acute sore throats but not conditions like diabetes mellitus type 2,345 (76.7%) and tuberculosis infection 386 (85.8%). Furthermore, the majority of the participants knew that OTC medications were not safe for use by the elderly 325 (72.2%), children 324 (72.0%) and pregnant 385 (85.6%). Details of these results are attached in Supplementary file 1.

Additionally, Table 2 shows the participants’ sources of OTC information and their level of importance. A substantial proportion, 179 (39.8%), found a college of pharmacy course, the Internet 149 (33.1%), and community pharmacists to be important OTC information sources. Additionally, 137 (30.4%) of the participants found the Internet to be an important source of OTC information to a high extent.

**Table 2:** Key sources of over-the-counter (OTC) information.

Source of information	Not at all *n* (%)	Minimally *n* (%)	Averagely *n* (%)	To high extent *n* (%)	Completely *n* (%)
College of pharmacy courses	34 (7.6)	43 (9.6%)	74 (16.4)	120 (26.7)	179 (39.8)
Books	46 (10.2)	68 (15.1)	135 (30.0)	108 (24.0)	93 (20.7)
Internet	28 (6.2)	46 (10.2)	90 (20.0)	137 (30.4)	149 (33.1)
Family and friends	100 (22.2)	135 (30.0)	115 (25.6)	52 (11.6)	48 (10.7)
Community pharmacists	44 (9.8)	59 (13.1)	95 (21.1)	119 (26.4)	133 (29.6)
Local workshops and conferences	92 (20.4)	100 (22.2)	106 (23.6)	85 (18.9)	67 (14.9)
International workshops and conferences	102 (22.7)	88 (19.6)	97 (21.6)	85 (18.9)	78 (17.3)

Table 3 shows the knowledge about various medications and their classification as OTC medications. The majority of the participants provided correct answers on medications considered OTC medications, with 324 (72.0%) citing Ibuprofen, 335 (74.4%) citing Paracetamol, 234 (52.0%) citing Cetirizine, and 238 (52.9%) citing Esomeprazole.

**Table 3:** Medication considered as over the counter (OTC) medications.

Medication	Categories	Number and percentage *n* (%)
Ibuprofen	Yes	324 (72.0)
	No	126 (28.0)
Insulin	Yes	179 (39.8)
	No	271 (60.2)
Penicillin	Yes	134 (29.8)
	No	316 (70.2)
Morphine	Yes	141 (31.3)
	No	309 (68.7)
Paracetamol	Yes	335 (74.4)
	No	115 (25.6)
Cetirizine	Yes	234 (52.0)
	No	216 (48.0)
Pantoprazole	Yes	236 (52.4)
	No	214 (47.6)
Loperamide	Yes	209 (46.4)
	No	241 (53.6)
Levothyroxine	Yes	135 (30.0)
	No	315 (70.0)
Esomeprazole	Yes	238 (52.9)
	No	212 (47.1)

### Attitude toward OTC medications

Table 4 shows the respondents’ attitudes toward the use of OTC medicine. Almost all the participants exhibited a positive attitude toward the use of OTC medication. Most of the participants agreed that OTC medications are cheaper and more convenient by 255 (56.7%) and that Paracetamol in overdose is a toxic agent 313 (69.5%). The majority, 290 (64.5%) of the participants, agreed that OTC medication can modify or alter the action of another drug and that not all OTC medication can be used in case of pregnancy 340 (75.6%). Most of them noted that OTC medications are affected by storage conditions such as temperature, moisture, and direct sunlight 353 (78.4%), with almost half 236 (52.5%) attesting that they sought medication safety from physicians before using them.

**Table 4:** Attitude of the participants to the use of OTC medicine.

Statement	Strongly disagree *n* (%)	Disagree *n* (%)	Neutral *n* (%)	Agree *n* (%)	Strongly agree *n* (%)
All OTC medications are safe and effective	84 (18.7)	86 (19.1)	150 (33.3)	105 (23.3)	25 (5.6)
Over-the-counter medications that are used for self-medication are safe.	51 (11.3)	88 (19.6)	160 (35.6)	118 (26.2)	33 (7.3)
Over-the-counter medications are cheaper and more convenient.	21 (4.7)	45 (10.0)	129 (28.7)	140 (31.1)	115 (25.6)
Paracetamol in overdose is a toxic agent.	32 (7.1)	31 (6.9)	74 (16.4)	104 (23.1)	209 (46.4)
Over-the-counter medications can modify or alter the action of another drug.	25 (5.6)	23 (5.1)	112 (24.9)	133 (29.6)	157 (34.9)
All over-the-counter medications can be used in case of pregnancy	256 (56.9)	84 (18.7)	69 (15.3)	19 (4.2)	22 (4.9)
Storage conditions like temperature, moisture, and direct sunlight do not affect over-the-counter medications.	284 (63.1)	69 (15.3)	57 (12.7)	20 (4.4)	20 (4.4)
Liquid OTC medications could be used when opened after 1 month.	121 (26.9)	91 (20.2)	163 (36.2)	48 (10.7)	27 (6.0)
Eye/ear OTC drops could be used after 1 month of opening.	161 (35.8)	103 (22.9)	119 (26.4)	42 (9.3)	25 (5.6)
It is better not to take OTC medications in case of gastric pain.	22 (4.9)	53 (11.8)	193 (42.9)	109 (24.2)	73 (16.2)
I usually seek over-the-counter medication safety from the physician before using them.	46 (10.2)	48 (10.7)	120 (26.7)	106 (23.6)	130 (28.9)

Table 5 presents the relationship between participants’ socio-demographic information and level of knowledge about over-the-counter (OTC) medication. The results established a statistically significant association between age, year of study and OTC medication course with *p*-values<0.05 (0.016*, 0.003* and 0.001*), respectively and the level of knowledge about OTC medication. There was no statistically significant association between gender and the level of knowledge about OTC medication (*p* > 0.05). The study results revealed that 280 (62.2%) participants had good knowledge about OTC medication, while 170 (37.8%) had poor knowledge.

**Table 5:** Association between demographic information and level of knowledge about over the counter (OTC) medication.

Variables	Category	Level of knowledge		
		Poor *n* (%)	Good *n* (%)	*p*-value
Age (years)	18–20	89 (52.4)	81 (47.6)	0.016*
	21–22	76 (49.7)	77 (50.3)	
	23–25	47 (39.5)	72 (60.5)	
	Over 25	2 (25.0)	6 (75.0)	
Gender	Male	101 (47.1)	114 (52.9)	0.273
	Female	114 (48.4)	121 (51.6)	
Year of Study	1st Year	41 (45.1)	50 (54.9)	0.003*
	2nd Year	26 (38.8)	41 (61.2)	
	3rd Year	17 (35.4)	31 (64.6)	
	4th Year	28 (30.1)	65 (69.9)	
	5th Year	19 (27.1)	51 (72.9)	
	6th Year	21 (25.9)	60 (74.1)	
OTC medication course	Yes	32 (13.7)	201 (86.3)	0.001*
	No	108 (49.8)	109 (50.2)	

*P < 0.05 indicates statistical significance.*

## Discussion

The study specifically focuses on College of Pharmacy students at Taif University. Among the 450 participants, a significant portion were between 18 and 20 years old, with nearly half being females. The study found that most participants demonstrated good knowledge about OTC medication, correctly identifying its definition, side effects, limitations, and specific medications classified as OTC. They also exhibited positive attitudes toward OTC medication, recognizing its affordability and convenience while being aware of potential risks like Paracetamol toxicity and drug interactions. The study also found that important sources of OTC information included college courses, the Internet, and community pharmacists. Statistical analysis revealed significant associations between age, year of study, having taken an OTC medication course, and the level of knowledge about OTC medication. While most participants showed good knowledge, areas were identified for improvement, particularly among specific demographic groups and aspects of OTC medication use.

The majority of pharmacy students at Taif University (85.6%) demonstrated a solid understanding of the definition of over-the-counter (OTC) medications. A similar study was conducted by Abdullah et al. among University and College Students in Brunei Darussalam. The questionnaire results are similar to this study, where more than half (53%) of the students had a knowledge score above 7, demonstrating a good understanding of OTC medicine (23). Bekele et al. research on the knowledge and practice of OTC medicine among pharmacy and medical students found that most (80%) participants had strong knowledge of OTC medicine (1). A similar study was conducted in 2020 among medical and pharmacy students at Qassim University, Buraydah, Saudi Arabia – the study found that almost all the participants (95%) had good knowledge of self-medication (24). This proficiency likely stems from an educational foundation deeply rooted in pharmacology and medication management courses integral to their curriculum. Beyond classroom learning, many pharmacy students work in internships or employment in pharmacy settings, where they regularly encounter OTC medications. This hands-on experience is crucial, as it allows students to apply and reinforce their theoretical knowledge, enhancing their understanding of OTC medications in a practical, real-world context. The study found that 88.2% of students were aware of the potential side effects associated with improper OTC medication use. The findings align with those of Mok et al.’s (3) study on the prevalence and perception of self-medication among adults in the Klang Valley, Malaysia, where the prevalence was 63.5%. Zafar et al. also found that prevalence and awareness of risks associated with self-medication among university students in Karachi, Pakistan, was as high as 76% (25). Also, Similar findings were reported in a study conducted among pharmacy students at Qassim University, where a majority demonstrated high awareness of OTC medication risks and benefits (26).

This understanding is emphasized extensively throughout pharmacy education, where students learn about the therapeutic effects of drugs and their potential adverse effects. This educational focus is supported by public health campaigns and media exposure, which frequently highlight the risks associated with medication misuse. These campaigns emphasize the importance of exercising caution when advising on or using OTC medications. Over half of the students (51.8%) reported having completed a course specifically focused on OTC medications, suggesting strong curricular support for this area of pharmacy education. This may reflect either a mandatory program component or elective courses aimed at broadening their knowledge base, particularly in community pharmacy and public health contexts. Personal interest in these areas might also motivate students to pursue additional learning opportunities to enhance their expertise in managing and counseling on OTC medications effectively. Similar results are mirrored in Ramadan et al.’s research on self-medication among undergraduate medical students at Alexandria University’s Faculty of Medicine. The self-medication rate was directly associated with level of education, being higher among those who had completed the 6 years of study (27). Education is, therefore, seen to influence self-medication among university students positively. The data indicates that most students (65.1%) understand the risks of using expired medications. In Saudi Arabia, prevalence is equally high, aligning this study’s results with the findings of Althagafi et al. study on the attitude and prevalence of self-medication among pharmacy students in King Abdulaziz University in Jeddah—over 67% of the participants thought that they possessed sufficient knowledge on OTC medication and potential side effects (28). A cross-sectional study in Saudi Arabia was conducted by Malli et al. and found that the prevalence of self-medication among college students was 55%, where education had no bearing on the use of OTC medication (29). However, medical students had a higher awareness compared to non-medical students. This awareness is instilled through specific safety training sessions in the pharmacy curriculum. These sessions emphasize the critical aspects of medication storage, handling, and regulatory compliance, including adherence to expiry dates. Such knowledge is essential for ensuring that medications provide the intended therapeutic effects and do not harm consumers. About half of the participants (51.6%) recognized that it is inappropriate to recommend medications based solely on personal experience. This understanding underscores the ethical training and legal education provided in pharmacy schools, which teach students about the responsibilities and potential legal repercussions of providing unprofessional advice. This foundation helps cultivate professional caution in medication referral practices, reinforcing the need for assessments based on sound medical and pharmacological principles rather than personal anecdotes. Students clearly understood the appropriate uses of OTC medications, recognizing their limitations in treating serious or chronic conditions. This awareness likely results from focused educational modules that discuss the scope and limitations of various medications, including OTC drugs. A cross-sectional study on the motive behind self-medication among by University Students in Saudi Arabia was conducted by Orayj et al. (26), where most students reported that the main cause of their use of OTC medication was a headache (35%), pain (21%), and fever (17%) – level of education and income were significant factors in the study. Clinical placements enhance this knowledge by providing real-life exposure where students can observe the consequences of medication choices and learn about the importance of appropriate drug use under the guidance of experienced professionals. The awareness among students regarding using OTC medications in vulnerable populations highlights the specialized training they receive in areas such as geriatric, pediatric, and maternity pharmacotherapy. These courses are designed to address such groups’ unique physiological considerations and medication sensitivity, ensuring that future pharmacists are well-prepared to advise on medication safety in a diverse patient population. According to Albert et al., nearly half of the adult population in America between the ages of 75 and 85 are regular users of OTC medication and recommended interventions to ensure that they receive training and education on safe self-medication. The key policy interventions recommended align with our recommendations—health literacy, OTC use and family care alongside utilizing technology to ensure optimal use of OTC medicines (30). Similar to this study’s correlation between level of education and knowledge about OTC medication and potential risks, we can conclude that existing medical literature points toward higher knowledge and awareness of OTC medication among people with higher levels of education, other demographic factors such as age and sex notwithstanding. Usage of OTC medicines among pregnant women who attended primary care facilities in Malang, Indonesia, showed that they were more knowledgeable about type and side effects of the medicines, compared to the general population (31). Their level of awareness and knowledge of OTC medicines also increased with increase in education level—the effect of correct medicines, dosage, and potential risks was statistically significant among women with high-school-level education or higher. The reliance on academic courses, the Internet, and community pharmacists for information on OTC medications illustrates the multifaceted approach to learning in pharmacy education. College courses provide a structured, comprehensive foundation, while the accessibility of digital resources offers Supplementary information that is readily available. Furthermore, interactions with community pharmacists during practical training or employment provide valuable real-world insights, allowing students to apply and expand their knowledge in actual pharmacy settings.

### Strengths and limitations

The study on pharmacy students’ views on OTC medication at Taif University has notable limitations and strengths. The snowball sampling method used may introduce sampling bias due to reliance on existing participants for recruitment, potentially leading to a non-representative sample. Additionally, self-reported data may be influenced by recall bias and social desirability bias, impacting result accuracy (32). Focusing solely on Taif University pharmacy students limits generalizability to other populations or settings. Despite these, strengths include a large sample size of 450 participants, enhancing statistical power and generalizability. Robust data collection methods provide nuanced insights, including demographic information and attitude assessment. Statistical analyses like chi-square tests improve validity. Ethical principles were adhered to, with approval from the university’s ethics committee, ensuring participant confidentiality and informed consent and enhancing credibility.

## Conclusion

The research at Taif University discovered that pharmacy students usually have a strong understanding of over-the-counter (OTC) medications, with advanced students and those who have taken OTC courses demonstrating better knowledge levels. However, there are gaps in safety standards, such as checking expiration dates and reading labels. Collaboration among medical experts is encouraged to increase awareness. The study identifies areas for educational improvement and provides important insights for future initiatives. It contributes to the current literature, leading future research and initiatives to fill knowledge gaps. Understanding student views is critical for healthcare practitioners because it allows them to improve pharmacy education and ensure that future pharmacists can advise on safe OTC usage, perhaps via specialized teaching modules.

## Data Availability

The raw data supporting the conclusions of this article will be made available by the authors, without undue reservation.
