# Supplementary material for: Knowledge and attitudes toward over-the-counter medications among pharmacy students: insights from a cross-sectional study in Taif University, Saudi Arabia
Source: Front Med (Lausanne). 2024 Nov 12;11:1435707. doi: 10.3389/fmed.2024.1435707 (PMC11588449; doi:10.3389/fmed.2024.1435707)
Supplement: Supplementary file 1 [file Table_1.DOCX]

**Supplementary file 1:** Participants’ knowledge of over-the-counter (OTC) medication

| **Questions** | **Categories** | **Frequency and Proportion n (%)** |
| --- | --- | --- |
| which of the following statements defines OTC medication properly? | Medication that can be bought only by prescription | 23 (5.1) |
|  | Medication that can be bought without a prescription | 42 (9.3) |
|  | Medication that can be sold to a consumer without a prescription from pharmacy personnel as compared to prescription medication, which is dispensed only to consumers possessing a valid prescription | 385 (85.6) |
| OTC medications could cause side effects when used improperly: | Strongly agree | 264 (58.7) |
|  | Agree | 133 (29.6) |
|  | Neutral | 38 (8.4) |
|  | Disagree | 12 (2.7) |
|  | Strongly disagree | 3 (0.7) |
| OTC medication are always used on the prescription of a doctor | Yes | 81 (18.0) |
|  | No | 323 (71.8) |
|  | I don’t know | 46 (10.2) |
| Have you taken a course specifically about OTC medications yet? | Yes | 233 (51.8) |
|  | No | 200 (44.4) |
|  | I don’t know | 17(3.8) |
| Two OTC medications containing the same active ingredients can be used together. | Strongly agree | 20 (4.4) |
|  | Agree | 25 (5.6) |
|  | Neutral | 84 (18.7) |
|  | Disagree | 129 (28.7) |
|  | Strongly disagree | 192 (42.7) |
| It is safe to use OTC medications past its expiration date | Strongly agree | 22 (4.9) |
|  | Agree | 19 (4.2) |
|  | Neutral | 40 (8.9) |
|  | Disagree | 76 (16.9) |
|  | Strongly disagree | 293 (65.1) |
| Can you recommend OTC medications to friends or family based on your personal use? | Yes | 171 (38.0) |
|  | No | 232 (51.6) |
|  | I don’t know | 47 (10.4) |
| Can OTC medications be harmful if misused? | Yes | 413 (91.8) |
|  | No | 15 (3.3) |
|  | I don’t know | 22 (4.9) |
| OTC medication are commonly used to treat condition like acute sore throat | Yes | 344 (76.4) |
|  | No | 106 (23.6) |
| OTC medication are commonly used to treat condition like diabetes mellitus type 2 | Yes | 105 (23.3) |
|  | No | 345 (76.7) |
| OTC medications are commonly used to treat condition like tuberculosis infection (TB) | Yes | 64 (14.2) |
|  | No | 386 (85.8) |
| OTC medication are commonly used to treat condition like infrequent constipation | Yes | 88 (19.6) |
|  | No | 362 (80.4) |
| OTC medications are likely safe for pregnancy | Yes | 65 (14.4) |
|  | No | 385 (85.6) |
| OTC medications are likely safe for elderly | Yes | 125 (27.8) |
|  | No | 325 (72.2) |
| OTC medications are likely safe for children | Yes | 126 (28.0) |
|  | No | 324 (72.0) |
| OTC medications are likely safe for adolescent/middle adults | Yes | 394 (87.6) |
|  | No | 56 (12.4) |
| If suspected side-effect(s) are seen, then one should: | Immediately stop using the drug | 150 (33.3) |
|  | Take low dose until side effect(s) subside | 29 (6.4) |
|  | Continue taking the medication regardless the side effects | 9 (2.0) |
|  | Report to a doctor or pharmacist | 262 (58.3) |
| All OTC, when taken along with prescribed medication, are safe. | Strongly agree | 17 (3.8) |
|  | Agree | 37 (8.2) |
|  | Neutral | 157 (34.9) |
|  | Disagree | 131 (29.1) |
|  | Strongly disagree | 108 (24.0) |
| OTC medications are approved for self-care. | Strongly agree | 108 (24.0) |
|  | Agree | 179 (39.8) |
|  | Neutral | 147 (32.7) |
|  | Disagree | 10 (2.2) |
|  | Strongly disagree | 6 (1.3) |
